# Beyond the first cut: evaluating CMV IHC reliability in serial gastrointestinal biopsy sections

**DOI:** 10.1007/s00428-025-04226-y

**Published:** 2025-09-11

**Authors:** Anna Hochner-Ger, Liat Anafi, Ido Veisman, Iris Barshack, Chen Mayer

**Affiliations:** 1 Institute of Pathology, Sheba Medical Center, Tel Hashomer, Ramat Gan, Israel; 2 Department of Gastroenterology, Sheba Medical Center, Tel Hashomer, Ramat Gan, Israel; 3 Faculty of Medicine, Tel-Aviv University, Tel Aviv, Israel

**Keywords:** CMV, Immunohistochemistry, Colitis, Serial sectioning

## Abstract

Cytomegalovirus(CMV) is a major pathogen in immunocompromised individuals, often causing severe gastrointestinal complications. Immunohistochemistry (IHC) is widely used for CMV detection due to its high specificity, but its reliability in cases with sparse CMV-positive cells remains uncertain. This study evaluates the reproducibility of CMV IHC in gastrointestinal biopsies by assessing CMV detection across serial sections. A prospective observational study was conducted at the Pathology Institute of Sheba Medical Center between the years 2023 and 2024. Twenty-five CMV colitis cases diagnosed based on IHC were included and categorized as positive (≥ 2 CMV-positive cells) or equivocal (1 CMV-positive cell). Serial sectioning of the tissue blocks and subsequent CMV IHC staining was performed. Descriptive statistical analysis, including the Friedman test and Wilcoxon signed-rank test, was used to assess variability in CMV detection. CMV-positive cell counts varied significantly across serial sections (*p* = 0.047, Friedman test). While some cases remained stable, others fluctuated, transitioning from positive to equivocal or negative. Among initially positive cases, 33.3% (7/21) had at least one negative section, and 33.3% (7/21) had at least one equivocal section. Equivocal cases were highly unstable, with all showing at least one negative section and one converting to positive. Pairwise Wilcoxon tests showed no consistent significant differences. CMV IHC exhibits variability in low-positive cases, leading to diagnostic transitions. Serial sectioning enhances confidence but does not fully eliminate variability. A multimodal approach integrating IHC with molecular methods and further standardization of IHC protocols may improve CMV detection accuracy.

## Introduction

Cytomegalovirus (CMV) is a significant pathogen in immunocompromised individuals, often causing severe gastrointestinal (GI) complications such as ulcers, bleeding, or perforation [1]. Accurate detection of CMV in GI biopsies is critical for timely diagnosis and effective management [2]. Immunohistochemical (IHC) staining for CMV is a widely used diagnostic method due to its high specificity and ability to localize viral antigens within infected cells [3]. By enabling the direct correlation of viral presence with tissue damage, IHC is a cornerstone of CMV diagnosis in pathology practice.

While CMV IHC is often regarded as the gold standard for histopathological diagnosis, its reliability, particularly in cases with sparse-positive cells, remains a subject of interest. Despite its high specificity, CMV IHC is less sensitive than molecular methods such as polymerase chain reaction (PCR) or in situ hybridization, and false-negative results can occur due to low viral loads or suboptimal staining protocols [3, 4]. Conversely, false positives, though rare, may arise from nonspecific staining or interpretative errors. These challenges are particularly pronounced in “low-positive” cases, where identifying a few CMV-positive cells can carry significant clinical implications, potentially influencing antiviral treatment decisions [5].

Recent studies have explored the role of IHC in CMV diagnosis, particularly in inflammatory bowel disease (IBD) patients, where CMV infection can complicate disease progression and management. In these cases, apoptotic bodies and crypt dropout have been associated with CMV infection, and even equivocal IHC staining may indicate true infection [6]. Additionally, comparative analyses of IHC and PCR suggest that while both methods are effective, IHC is particularly useful for detecting CMV in biopsies from ulcer bases and edges, whereas PCR may identify additional cases that could benefit from antiviral therapy [4].

In clinical practice, variability in factors such as tissue fixation, antigen retrieval, staining protocols, and antibody performance can further impact the consistency and reproducibility of CMV IHC results. Studies have also shown that severe inflammation and the number of tissue blocks examined may impact CMV detection rates [7]. Furthermore, interobserver variability plays a role in diagnostic accuracy, particularly when distinguishing true CMV inclusions from mimicking artifacts. These issues highlight the need for a systematic evaluation of CMV IHC reliability to better understand its limitations and refine its use as a diagnostic tool.

This study aims to assess the reliability of CMV immunostaining in GI biopsies, specifically in the context of diagnosing CMV colitis. By examining staining patterns across deeper sections from the same tissue blocks in cases with CMV positivity, we seek to evaluate the consistency of IHC as a diagnostic tool. Our findings will provide valuable insights into the reproducibility of CMV detection and its implications for improving diagnostic accuracy and clinical decision-making in cases of suspected CMV colitis.

## Methods

This prospective observational study was conducted at the Pathology Institute of Sheba Medical Center between 2023 and 2024. The study aimed to assess the variability of CMV detection in colonic biopsies and resection specimens through serial sectioning and repeat immunohistochemical (IHC) staining.

### Case selection

All cases diagnosed with CMV colitis based on IHC were included if they met one of two criteria: (1) CMV-positive, defined as at least two CMV-infected cells identified in the initial stained section or (2) equivocal, defined as only a single CMV-infected cell. Cases were excluded if tissue blocks were unavailable, insufficient material remained for further sectioning, or the initial IHC slide demonstrated more than 25 CMV-positive cells, as quantification in such cases was considered less meaningful.

### Histological analysis and serial sectioning

For each included case, the original formalin-fixed paraffin-embedded (FFPE) tissue block was serially sectioned to obtain six consecutive 4-µm-thick sections. Each section was re-stained with IHC for CMV to evaluate the distribution and reproducibility of CMV detection across different levels of the same tissue sample.

### Immunohistochemistry

IHC staining was performed using a standardized protocol with a commercially available monoclonal antibody (Dako, Carpinteria, CA; diluted 1:25). The procedure followed routine laboratory protocols, including deparaffinization, antigen retrieval, blocking, primary antibody incubation, secondary antibody application, and chromogen detection. Positive and negative controls were included in each staining run.

### CMV-positive cell identification

**Fig. 1:**
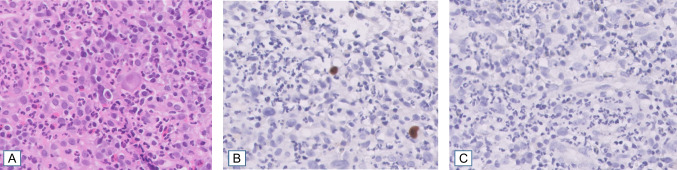
Serial sections from the same area of a colonic biopsy block. (**A**) Hematoxylin and eosin (H&E) stain of the region of interest. (**B**) CMV immunohistochemical stain on a subsequent section showing two CMVpositive stromal cell nuclei. (**C**) A deeper IHC-stained section of the same area showing no detectable CMV-positive cells

A certified gastrointestinal (GI) pathologist reviewed all stained sections. CMV-positive cells were identified by distinct nuclear staining, characterized by fully stained nuclear contours, the presence of nucleoli or macronucleoli, and the absence of isolated cytoplasmic staining without nuclear involvement. The number of CMV-positive cells in each of the seven sections (initial and six additional sections) was recorded. Figure 1 presents a representative example of a positively stained CMV-infected nucleus, as observed in diagnostic sections.

### Digital scanning

Hematoxylin and eosin (H&E) and CMV IHC-stained slides were digitally scanned using the Philips IntelliSite scanner, allowing for high-resolution digital analysis and standardized cell counting across serial sections.

### Data collection and analysis

For each case, the number of CMV-positive cells was documented in a structured table by a senior pathologist. Descriptive statistical analysis was performed to assess variability in CMV detection across serial sections. The study aimed to evaluate the reliability of CMV detection from a single tissue section and determine whether additional sectioning improves diagnostic confidence, particularly in borderline cases. Generative AI tools were used in creating the figures.

## Results

**Fig. 2:**
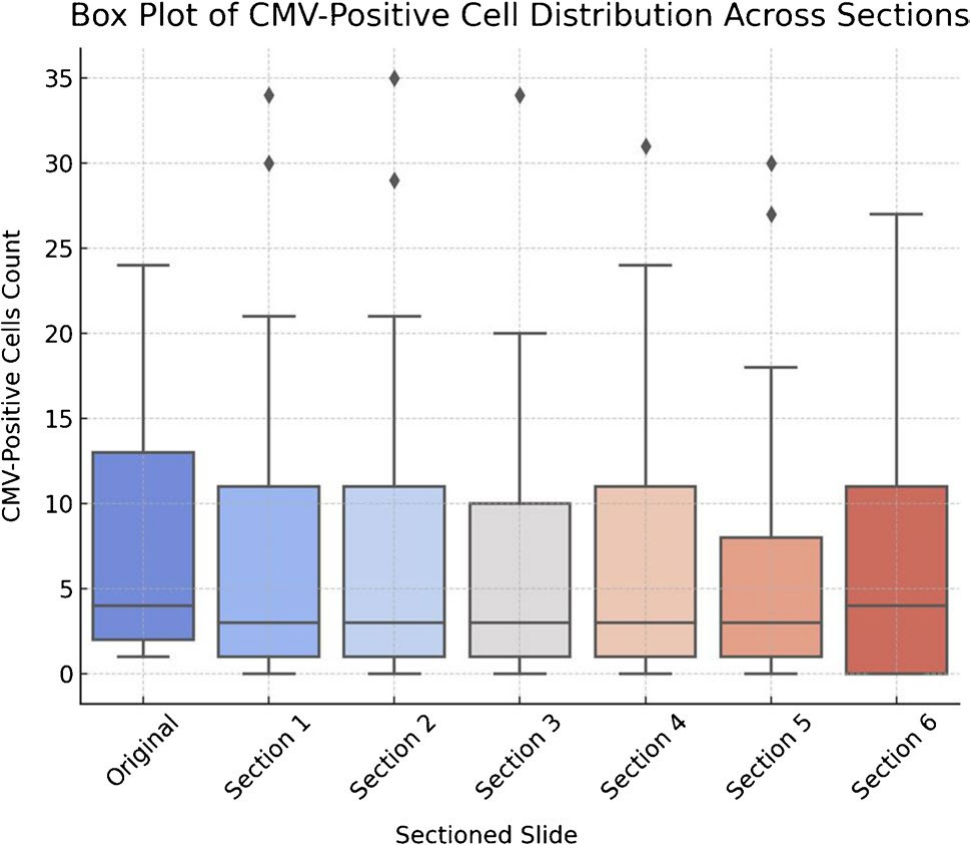
Boxplot showing the distribution of CMV-positive cell counts across serial sections

A total of 25 cases were analyzed, with CMV-positive cell counts recorded across serial sections for each case. Of these, 21 cases were classified as positive in the initial section, defined as having at least two CMV-positive cells, while four cases were classified as equivocal, defined as having a single CMV-positive cell. Across deeper sections, CMV detection exhibited substantial variability, with some cases maintaining a consistent number of CMV-positive cells while others showed fluctuations, including cases that transitioned to equivocal or negative classifications in at least one deeper section. The median CMV-positive cell count in the initial section was 8 (IQR, 2–15), with a gradual decline in median counts across deeper sections, reaching a median of 7 (IQR, 1–14) by the final section. Figure 2 illustrates the distribution of CMV-positive cells across serial sections.

### Variability of CMV-positive cells across serial sections

To evaluate whether CMV detection varied significantly across different section levels, a Friedman test was performed, revealing a statistically significant difference in CMV-positive cell counts across serial sections (*p* = 0.047). Given the non-parametric nature of the data, a one-way repeated measures ANOVA was not applicable due to violations of normality assumptions based on Shapiro–Wilk tests conducted for each section. Pairwise comparisons between the original section and each deeper section using the Wilcoxon signed-rank test did not reveal a consistent significant difference, though fluctuations in CMV detection across sections suggest a potential impact of sectioning variability on diagnostic confidence. The presence or absence of CMV-positive cells in deeper sections appeared inconsistent in certain cases, emphasizing the importance of serial sectioning in low-positive cases.

### Diagnostic transitions across serial sections

**Fig. 3:**
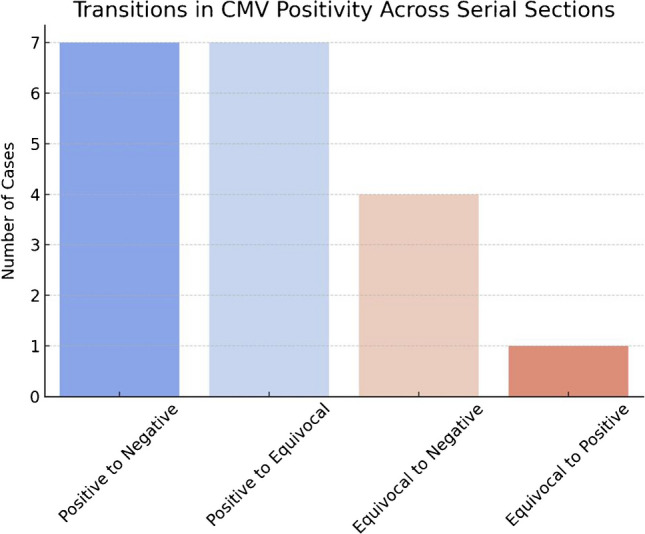
Bar plot illustrating diagnostic transitions across serial sections

Changes in diagnostic classification were observed in a substantial proportion of cases upon deeper sectioning. Seven cases originally classified as positive transitioned to negative in at least one deeper section, while seven additional positive cases transitioned to equivocal in at least one deeper section. Among the four cases initially classified as equivocal, four transitioned to negative in at least one deeper section, while one transitioned to positive with at least two CMV-positive cells in a deeper section. These findings suggest that while initially positive cases generally retained at least some level of positivity, the classification of equivocal cases was less stable, with a greater likelihood of diagnostic fluctuation. Figure 3 illustrates the frequency of these transitions in a bar plot.

**Fig. 4:**
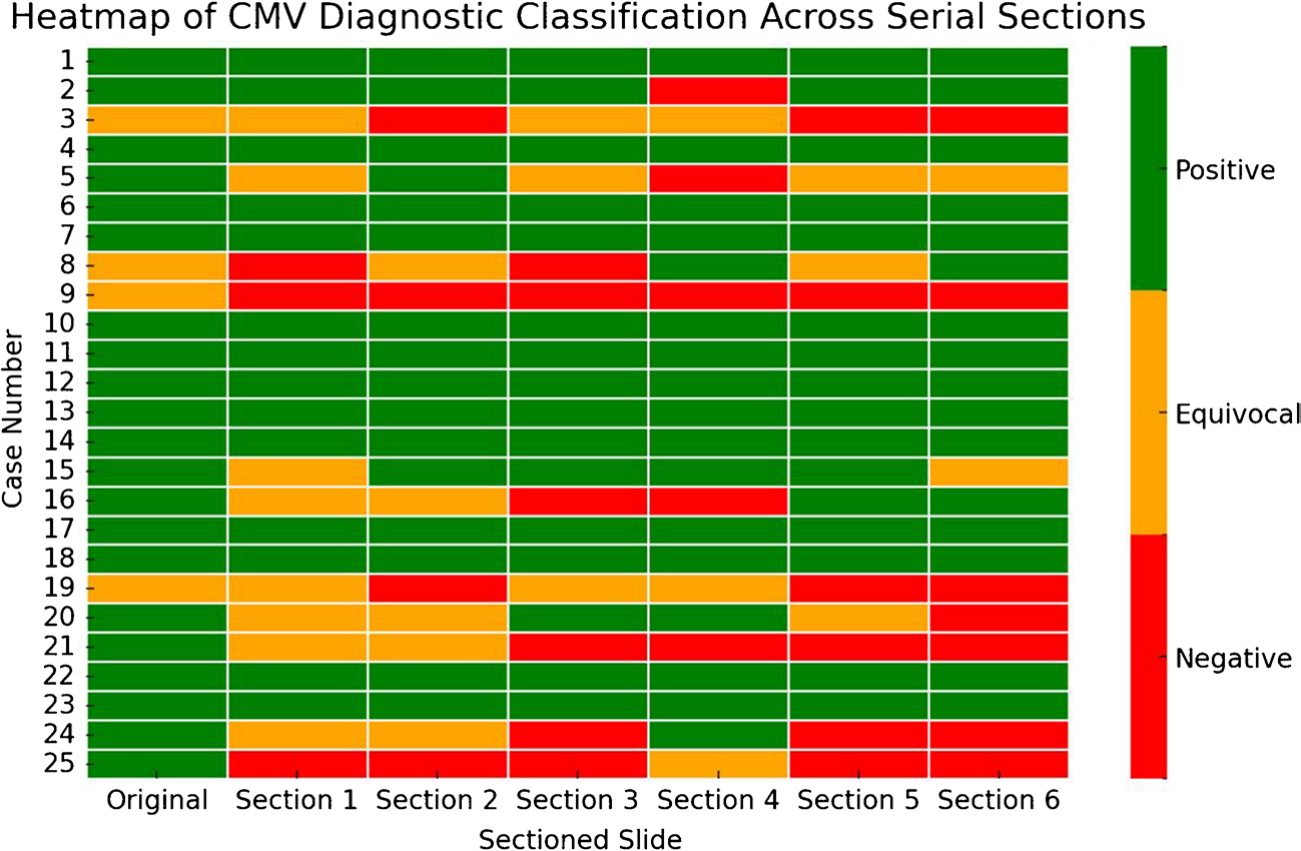
Heatmap showing categorical classifications across serial sections

To further visualize diagnostic variability across sections, a categorical heatmap was created to represent negative, equivocal, and positive classifications in each section for every case (Fig. 4). Negative cases (0 positive cells) are represented in red, equivocal cases (1 positive cell) in orange and positive cases (≥ 2 positive cells) in green. This visualization highlights how CMV detection fluctuates across deeper sections, with multiple cases transitioning between diagnostic categories.

## Discussion

The findings of this study highlight important considerations regarding the reliability of CMV immunohistochemistry (IHC) in gastrointestinal biopsies. Our analysis of serial sections from CMV-positive and equivocal cases underscores key limitations of CMV IHC, particularly in cases with low viral burden, and highlight the diagnostic challenges posed by sampling variability, interobserver discrepancies, and the inherent sensitivity constraints of IHC when compared to molecular methods.

A primary observation in this study is the variability in CMV detection across serial sections. While some cases maintained a relatively stable number of CMV-positive cells across deeper sections, others exhibited significant fluctuations, with CMV-positive cells appearing in some sections and being absent in others. This variability suggests that CMV-infected cells may be sparsely distributed within tissue samples, and their detection can be influenced by the specific level of sectioning. This phenomenon aligns with prior reports indicating that CMV IHC, while highly specific, may suffer from reduced sensitivity, particularly in cases with low viral loads. Such limitations are of clinical significance, as failure to detect CMV in a single tissue section may lead to false-negative diagnoses and delayed antiviral therapy initiation.

The analysis of equivocal cases, defined as those with a single CMV-positive cell in the initial section, further highlights the challenges associated with CMV IHC interpretation. In a subset of these cases, serial sectioning confirmed additional CMV-positive cells, supporting the presence of active infection. However, in other cases, deeper sections failed to reveal further CMV positivity, raising concerns about the reproducibility of a single CMV-positive cell as definitive evidence of infection. These findings suggest that serial sectioning may provide incremental diagnostic confidence in borderline cases, potentially reducing false-positive or false-negative results. Given the clinical implications of CMV detection in patients with inflammatory bowel disease or other immunocompromised states, the findings emphasize the need for a cautious approach when interpreting equivocal IHC results.

Another critical factor in CMV IHC reliability is interobserver variability. Although our study utilized digital slide scanning and a single observer to standardize cell counting, the interpretation of CMV IHC remains inherently subjective. Small CMV inclusions, apoptotic bodies, and nonspecific staining can pose challenges in accurately identifying infected cells, particularly in inflamed or necrotic tissue. Standardization of CMV IHC criteria, along with training initiatives aimed at improving pathologist agreement, may help mitigate these inconsistencies and enhance diagnostic accuracy.

These findings gain additional relevance when considered in light of current clinical guidelines for CMV detection in inflammatory bowel disease. CMV is a common opportunistic infection in severe, treatment-refractory IBD—especially ulcerative colitis—and is associated with poorer outcomes, prompting guideline recommendations for early evaluation. The recent American College of Gastroenterology (ACG) update advises that all hospitalized patients with acute severe UC undergo early endoscopy with colonic biopsies to assess for CMV colitis [8]. Similarly, the European Crohn’s and Colitis Organization (ECCO) consensus recommends screening for CMV in any IBD patient who is not improving on immunosuppressive therapy [9]. According to ECCO, diagnostic confirmation of CMV colitis should rely on tissue-based testing during endoscopy, with quantitative PCR and immunohistochemistry (IHC) on colonic biopsies recommended due to their higher sensitivity compared to H&E staining. Biopsies should target areas of deep ulceration, where viral load is typically highest, and multiple sites may be required in Crohn’s disease due to its patchy nature. These recommendations underscore the need for robust and reproducible tissue-based CMV detection methods, particularly in low-positivity scenarios.

While IHC remains advantageous in providing spatial and morphologic context by allowing direct visualization of CMV-infected cells within the affected tissue, its sensitivity is inherently lower than that of PCR-based methods, which can detect viral DNA even in cases with minimal histopathologic evidence of infection [4]. This discrepancy is particularly relevant in cases where CMV involvement is suspected but not histologically overt. However, the higher sensitivity of PCR must be interpreted with caution, as it may detect low-level CMV DNA that lacks clinical significance, potentially leading to overtreatment in certain scenarios. A combined diagnostic approach utilizing both IHC and molecular techniques may offer the most robust strategy for CMV detection, particularly in diagnostically challenging cases.

This study has several limitations that should be considered. First, the sample size was limited to positive or equivocal cases, which may introduce selection bias, as “negative” cases may also present positivity on deeper sections. Additionally, the study focused exclusively on CMV colitis, and the findings may not be generalizable to CMV infections in other sites. Future research should explore larger, multicenter studies to validate our observations and further assess the impact of different staining protocols, antibody clones, and tissue processing techniques on CMV IHC reliability.

## Conclusion

The results of this study underscore the diagnostic limitations of CMV IHC in GI biopsies, particularly in cases with low viral burden. Serial sectioning may improve diagnostic confidence in equivocal cases by identifying additional CMV-positive cells. Given the potential for sampling bias and interobserver variability, a multimodal diagnostic approach integrating IHC with molecular methods such as PCR may provide the most comprehensive strategy for CMV detection. Continued efforts to optimize IHC protocols and establish standardized diagnostic criteria are essential to improving the reliability and clinical applicability of CMV IHC in routine pathology practice.

## Data Availability

The data that supports the findings of this study are available from the corresponding author (CM), upon reasonable request.

## Declarations

### Permission to reproduce material from other sources

No previously published material was reproduced in this manuscript.
